# Rubber Tube–Based Triboelectric Nanogenerator for Simultaneous Energy Harvesting and Real‐Time Health Monitoring in Taekwondo Athletes

**DOI:** 10.1002/open.202500454

**Published:** 2026-03-25

**Authors:** Chengquan Piao, Jianzhong Wan, Qichen Mu, Peiying Jia

**Affiliations:** ^1^ Foundation I Qilu Institute of Technology Jinan China; ^2^ Physical Education Institute Sichuan University of Arts and Science Dazhou China; ^3^ School of Humanities Communication University of Shanxi Jinzhong China

**Keywords:** human motion, rubber tube, self‐powered sensing, triboelectric nanogenerators

## Abstract

Recent advances in wearable sensor technologies and functional material engineering have significantly accelerated the development of intelligent sports systems. Here, we developed a rubber tube–structured triboelectric nanogenerator (RT‐TENG) that integrates the triboelectric layer, conductive electrode, and mechanical support into a single body, offering simplified fabrication and reduced cost while maintaining reliable output. By employing fluorinated ethylene propylene (FEP) and as the triboelectric pair, the RT‐TENG achieves a peak output of 133 V, 27 μA, and 52 nC, with a maximum power of ≈1.0 mW under optimal load. It exhibits strong environmental adaptability with temperature‐enhanced and humidity‐sensitive performance, as well as force‐dependent output and robust energy storage capability. Furthermore, the RT‐TENG successfully powers a digital thermo‐hygrometer and enables real‐time, self‐powered monitoring of joint motion and impact intensity in taekwondo, highlighting its promise for wearable biomechanical sensing and low‐power sports electronics.

## Graphical abstract

A flexible P(AA‐MA)‐PVA hydrogel‐based triboelectric nanogenerator (PP‐TENG) efficiently harvests biomechanical energy and enables self‐powered tennis motion sensing. The device integrates high stretchability, self‐healing, and polydimethylsiloxane protection, achieving 182 V output and real‐time multi‐joint monitoring for intelligent sports analysis and injury prevention.

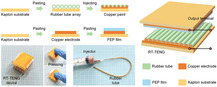

## 1 Introduction

Flexible electronics integrate functional materials onto deformable substrates, endowing sensors with superior conformability and dynamic responsiveness [1, 2]. This technological advancement underpins the expanding application of wearable devices across healthcare [3], biomedical monitoring [4], and intelligent sports [5]. In recent years, a diverse array of novel flexible materials has emerged, including smart textiles with hierarchical fiber architectures and high breathability [6], hydrogels with intrinsic stretchability and conductivity [7], and electrospun nanofibrous films characterized by large specific surface areas and tunable microstructures [8]. In detail, smart textiles, leveraging their three‐dimensional fiber networks, offer excellent scalability, mechanical compliance, and wearability, enabling continuous monitoring of joint kinematics and body posture [9]. Hydrogels exhibit outstanding skin conformability and environmental tolerance, making them ideal for epidermal bio‐signal detection and skin–device interfaces [10]. Electrospun films, due to their fine nanostructures, provide exceptional responsiveness to subtle mechanical stimuli and have been effectively employed in complex physical activities such as taekwondo for motion recognition, impact intensity assessment, and real‐time training feedback—enhancing the precision and interactivity of next‐generation sports systems [11]. Despite recent progress, wearable electronics still face challenges in mechanical durability, miniaturization, and sustainable energy supply [12, 13]. Conventional power limits wearable systems in dynamic settings, while self‐powered sensors with integrated energy harvesting offer a path toward autonomous, wireless sensing [14, 15].

Triboelectric nanogenerator (TENG) technology, proposal by Wang Group in 2012, is a novel energy conversion device based on the coupling effect of contact electrification and electrostatic induction, which can efficiently convert weak mechanical energy into electrical energy [14, 16, 17, 18, 19, 20, 21, 22, 23, 24, 25, 26, 27, 28, 29, 30, 31, 32, 33, 34, 35, 36, 37, 38, 39, 40, 41, 42, 43, 44]. Triboelectric nanogenerators operate by generating interfacial charges during repeated contact and separation of dissimilar materials, which induces an electric potential and drives alternating current through an external circuit [45, 46]. Owning to its simple structure, strong material adaptability, and low cost, TENG has shown great potential in the field of distributed micro energy harvesting, including micro wind energy [47, 48], water wave energy [49, 50], human motion energy [51, 52, 53], and various mechanical equipment vibration energy [54, 55]. It is worth noting that by integrating TENG into scenes such as carpets, clothing, insoles, and so on, low‐frequency vibration energy such as gait and joint bending can be efficiently captured [56, 57]. In addition to energy harvesting, TENGs offer high‐sensitivity mechanical signal detection without external power, enabling applications in tactile sensing, motion monitoring, and physiological signal acquisition [58, 59, 60, 61]. Recent efforts focus on integrating conductive hydrogels, electrospun nanofibers, MXene, and carbon quantum dots to enhance conductivity, stretchability, and environmental adaptability for wearable use [62, 63, 64, 65]. In dynamic scenarios like intelligent sports, self‐powered triboelectric sensors enable real‐time monitoring of movement intensity, posture, and impacts, supporting health evaluation, training optimization, and human–machine interaction [66, 67, 68]. With advances in materials, microstructure engineering, and system integration, TENGs are poised to play a key role in next‐generation low‐power, flexible sensing systems.

In this work, we proposed a rubber tube structure triboelectric nanogenerator (RT‐TENG) for bio‐mechanical energy harvesting and taekwondo sports monitoring. Unlike conventional layered configurations, the tube structure integrates the triboelectric layer, conductive electrode, and mechanical support into a unified body, effectively simplifying device fabrication and reducing production costs while maintaining stable electrical performance. Fluorinated ethylene propylene (FEP), with its strong electron affinity and excellent dielectric properties, is employed as the negative triboelectric layer, while functions as the positive counterpart. Systematic evaluation reveals that the RT‐TENG displays temperature‐enhanced output and humidity‐induced attenuation, highlighting its environmental adaptability and potential utility in self‐powered sensing systems. The RT‐TENG exhibits stable and repeatable electrical output under cyclic operation, with a peak open‐circuit voltage (*V*_OC_) of 133 V, short‐circuit current (*I*_SC_) of 27 μA, and transfer charge (*Q*_SC_) of 52 nC, while delivering a maximum output power of ∼1.0 mW at an optimal load of 3 MΩ, confirming its potential for efficient energy harvesting. The RT‐TENG exhibits force‐dependent electrical output, mechanical durability, and efficient energy storage capabilities, and successfully drives a commercial digital thermo‐hygrometer, demonstrating its potential for practical self‐powered sensing and low‐power electronics. The RT‐TENG enables self‐powered, high‐fidelity monitoring of joint motion, angular deformation, and impact intensity during taekwondo training, demonstrating its potential as a flexible wearable platform for real‐time biomechanical sensing in high‐intensity sports.

## 2 Experiments

### 2.1 Preparation of RT‐TENG Device

**FIGURE 1:**
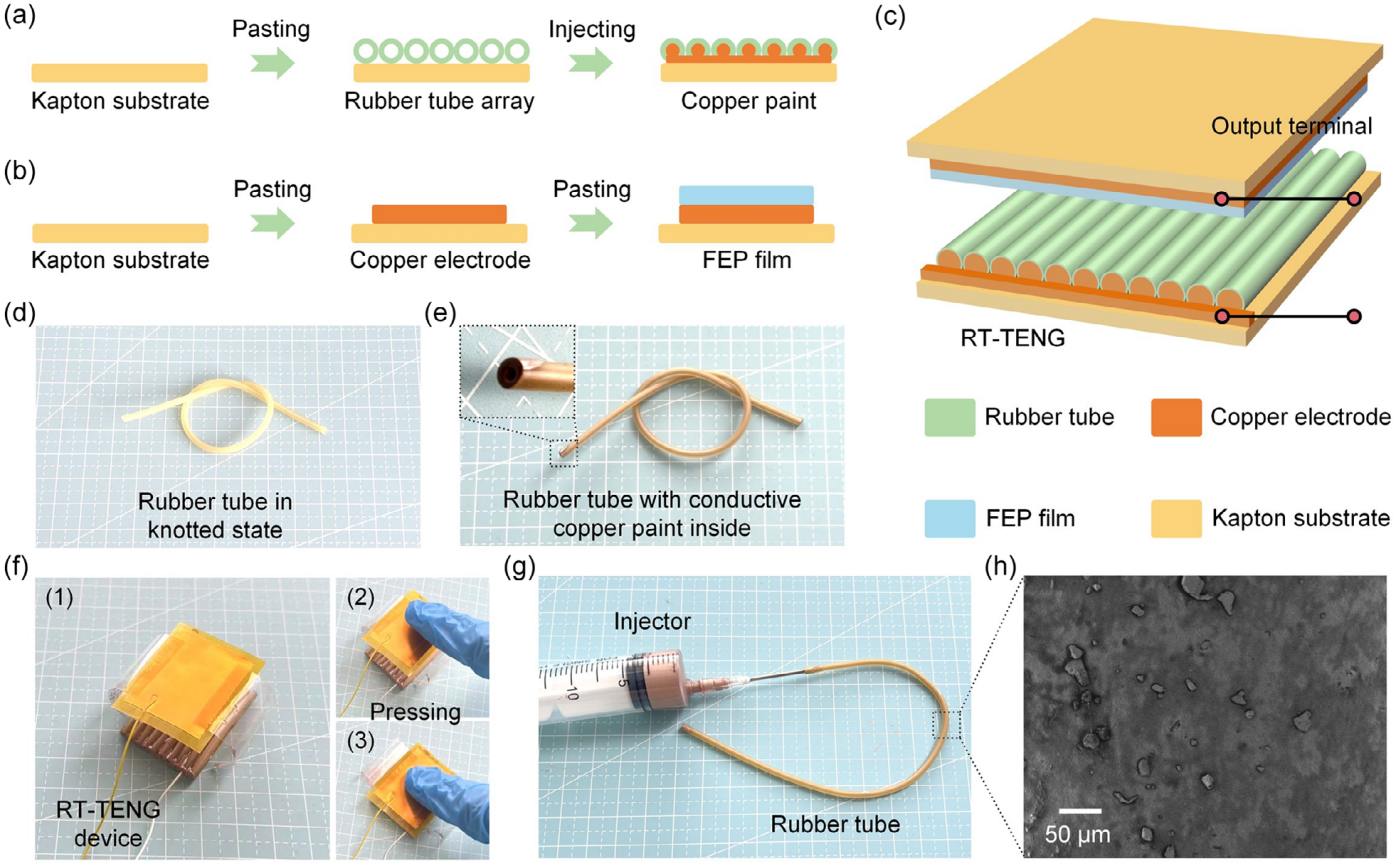
Structural design and fabrication of the RT‐TENG. (a) Schematic illustration of the bottom layer composed of hollow tubes filled with copper paint on a Kapton substrate. (b) Fabrication of the top layer consisting of a Cu electrode and laminated FEP film. (c) Assembly of the RT‐TENG with a compressible tube–film interface. (d) A rubber tube tied into a knot to demonstrate high flexibility. (e) Copper paint injected into the hollow core of the tube; inset shows the cross‐sectional view with internal copper coating. (f1–f3) Manual pressing of the assembled RT‐TENG, showing its softness and deformability under mechanical load. (g) Injection of copper paint into the tube using a syringe. (h) SEM image of the outer surface of the tube.

The fabrication process of the RT‐TENG is schematically illustrated in Figure 1a–c. As shown in Figure 1a, the bottom layer is prepared by first attaching an array of elastic tubes onto a flexible Kapton substrate. These tubes serve both as triboelectric materials and as a compressible microstructured interface. To enable electrical conductivity, copper paint is injected into the hollow interiors of the tubes using a syringe, forming embedded electrodes inside each tube. Figure 1b shows the construction of the top layer. A copper electrode is adhered onto another Kapton film to provide mechanical support and electrical conduction. Subsequently, the FEP film is laminated over the copper electrode. Owing to its strong electronegativity and low surface energy, the FEP film functions as an effective triboelectric material for charge generation. Finally, the complete device structure is assembled as illustrated in Figure 1c. The top and bottom layers are stacked face‐to‐face, forming a sandwich structure where the tube array is positioned directly beneath the FEP layer. The contact–separation between the tubes and FEP film during mechanical deformation leads to efficient triboelectric charge generation. The embedded copper paint inside the tubes and the top copper electrode serve as the two electrical terminals for energy output. This modular, compressible, and stretchable architecture enables the RT‐TENG to effectively convert biomechanical or environmental motion into electricity, offering advantages in flexibility, tunability, and ease of integration. The rubber tube used as the triboelectric material and structural support in the RT‐TENG is made from chloroprene rubber. This material exhibits a tensile strain of approximately 200% and an elastic modulus of 2 MPa. The operating temperature range of the rubber tube is from −40°C to 120°C, ensuring stable performance in a variety of environmental conditions. This material was chosen due to its excellent flexibility, mechanical durability, and moderate temperature resistance, which are critical for the dynamic deformation required in the RT‐TENG device.

### 2.2 Characterization and Measurements

Figure 1d shows a rubber tube manually tied into a knot, indicating its high flexibility and resistance to mechanical deformation. In Figure 1e, the interior of the tube is filled with conductive copper paint. The inset highlights the cross‐sectional view, where a uniform copper coating can be observed along the inner wall of the tube. Figure 1g illustrates the injection process using a standard syringe, where the copper paint is introduced into the hollow rubber tube. The injection nozzle is closely connected to one end of the tube to ensure smooth and complete filling. Figure 1f1 displays the assembled RT‐TENG device, consisting of stacked layers based on the tube array. In Figure 1f2, a finger presses down on the device to simulate mechanical input. Figure 1f3 shows the device under compression, with visible deformation indicating effective contact between triboelectric layers during the pressing action. Figure 1g illustrates the injection process of copper paint into the hollow rubber tube using a standard medical‐grade syringe. The nozzle of the syringe is tightly inserted into one end of the tube to ensure a smooth and continuous filling process. This simple yet effective method allows the formation of an embedded conductive layer along the inner surface of the tube, serving as the bottom electrode of the RT‐TENG. The flexibility of the tube ensures uniform distribution of the conductive medium without mechanical damage or leakage. To examine the surface morphology of the triboelectric layer, Figure 1h presents a scanning electron microscopy image of the outer surface of the rubber tube. The image reveals a non‐uniform, microscopically rough texture, which is favorable for increasing the effective contact area and enhancing interfacial friction during device operation. Such microstructural features can facilitate improved triboelectric charge generation by promoting stronger contact electrification between the rubber tube and the FEP film. The output performance of the TENG, including *V*_OC_, *I*_SC_, and *Q*_SC_, was measured using a Keithley 6514 electrometer operating in high‐impedance mode to ensure accurate signal acquisition with minimal distortion. A linear motor was employed to generate controlled and repeatable contact–separation motion. A force sensor was positioned beneath the TENG to monitor real‐time contact pressure, allowing dynamic adjustment of the motor's actuation to maintain consistent excitation conditions.

## 3 Results and Discussion

### 3.1 The Working Mechanism of RT‐TENG Device

**FIGURE 2:**
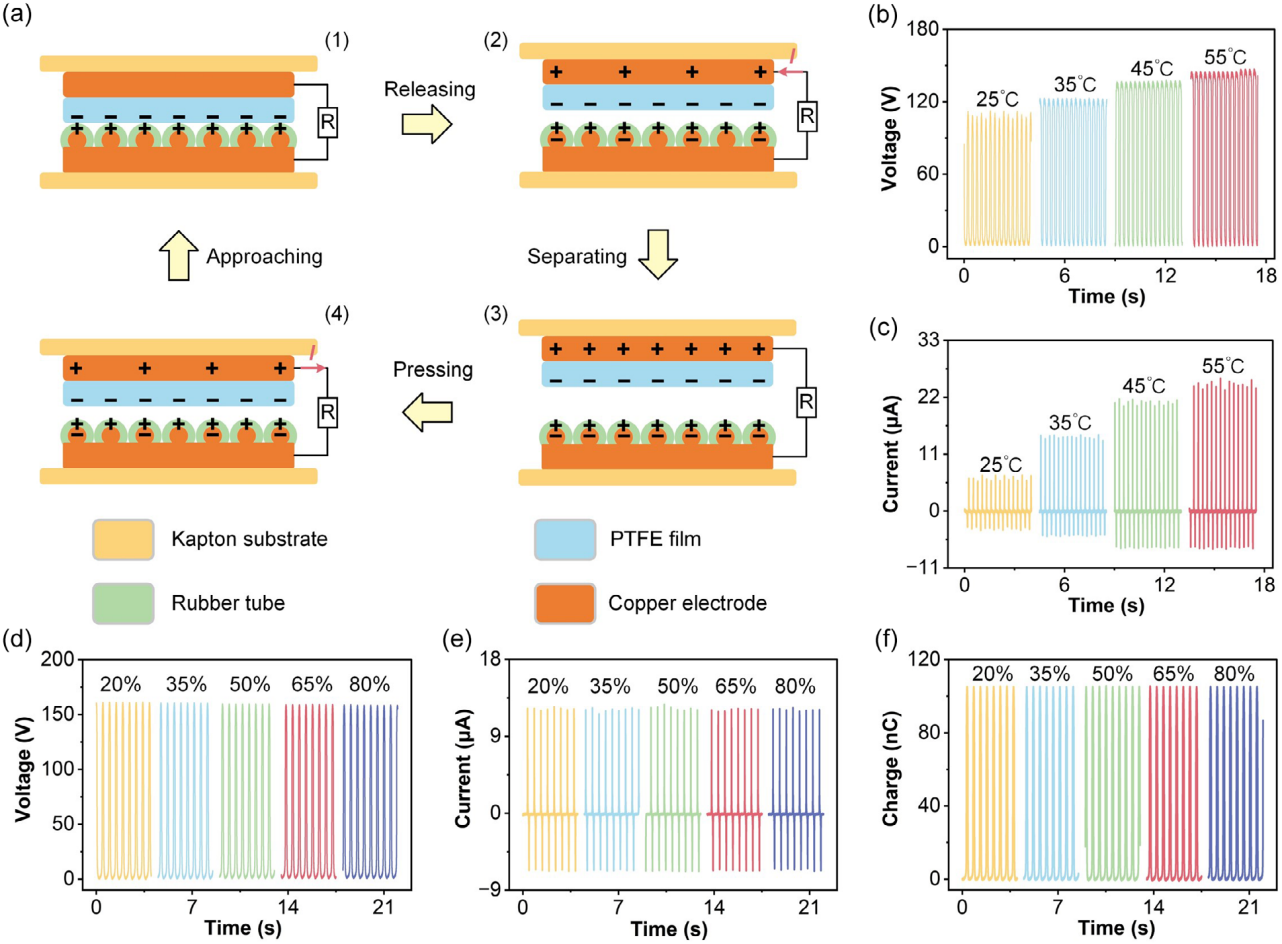
Working mechanism and environmental output performance of the RT‐TENG. (a1–a4) Schematic illustrations of the working mechanism based on contact–separation mode. The (b) *V*_OC_ and (c) *I*_SC_ of the RT‐TENG measured under different temperatures (25°C, 35°C, 45°C, and 55°C). The (d) *V*_OC_ (e) *I*_SC_ (f) *Q*_SC_ of the RT‐TENG under various RH levels (20%, 35%, 50%, 65%, and 80%).

The working principle of the RT‐TENG is illustrated in Figure 2a, based on the contact–separation mode between the FEP film and the tube array. During the initial state (Figure 2a1), the FEP film and the tubes are in full contact under external pressure. Due to the difference in electron affinity between the two materials, triboelectric charges are generated at the interface: negative charges accumulate on the FEP surface, while positive charges form on the tubes. At this point, no current flows through the external circuit since there is no potential difference. As the device begins to release and the two layers gradually separate (Figure 2a2), the electrostatic equilibrium is disturbed. The separation of oppositely charged surfaces creates a potential difference between the top and bottom electrodes. Driven by this potential, electrons are induced to flow through the external circuit from the bottom copper electrode (inside the tubes) to the top electrode, generating a transient current. In the fully separated state (Figure 2a3), the maximum potential difference is established due to the increased gap between the FEP film and the tube array. The induced charges on the electrodes are balanced by the static triboelectric charges on the contacting surfaces. When external force is reapplied (Figure 2a4), the two surfaces approach each other again. The potential difference reverses, causing electrons to flow in the opposite direction through the external circuit. This back‐and‐forth motion generates an alternating current output. This cyclic contact–separation process converts mechanical energy into electrical energy through triboelectrification and electrostatic induction. The elasticity of the tubes allows efficient charge generation and recovery, while the embedded copper paint ensures effective charge collection throughout the cycle.

### 3.2 Temperature and Humidity Dependence of RT‐TENG Performance

The electrical output performance of the RT‐TENG under varying environmental conditions is systematically evaluated in Figure 2b–f. Figure 2b shows the *V*_OC_ of the device at different ambient temperatures ranging from 25°C to 55°C. As the temperature increases, the output voltage also increases, indicating enhanced triboelectric charge generation. This may be attributed to increased molecular mobility and improved surface contact at elevated temperatures, which facilitate more effective charge transfer. Corresponding *I*_SC_ results are displayed in Figure 2c, showing a similar increasing trend with temperature. The current output rises from approximately 6.9 μA at 25°C to over 25.6 μA at 55°C, confirming that higher temperatures promote more dynamic charge flow and reduced internal resistance during contact–separation cycles. The electrical output performance of the RT‐TENG was also evaluated under varying relative humidity (RH) levels, ranging from 20% to 80%. As shown in Figure 2d–f, we observe a gradual decrease in the *V*_OC_, *I*_SC_, and *Q*_SC_ with increasing humidity. This decrease can be attributed to the adsorption of water molecules on the surface of the FEP and rubber tube materials. Mechanistically, water molecules adsorbed onto the surface of the triboelectric materials can interfere with the charge accumulation process. The presence of water molecules at the interface reduces the effective contact electrification between the FEP film and the rubber tube, as water molecules can act as a screen or barrier, reducing the efficiency of charge transfer. Additionally, water molecules may affect the surface polarity and decrease the electron affinity of FEP, thus lowering its ability to gain negative charge during contact. As humidity increases, the thickness of the water layer on the surface increases, further diminishing the interfacial friction and charge generation efficiency. This phenomenon highlights the sensitivity of the RT‐TENG to environmental conditions, with water molecules potentially influencing both the mechanical and electrical characteristics of the device during operation under high humidity.

### 3.3 Electrical Output Characteristics and Energy Harvesting Capability of the RT‐TENG Device

**FIGURE 3:**
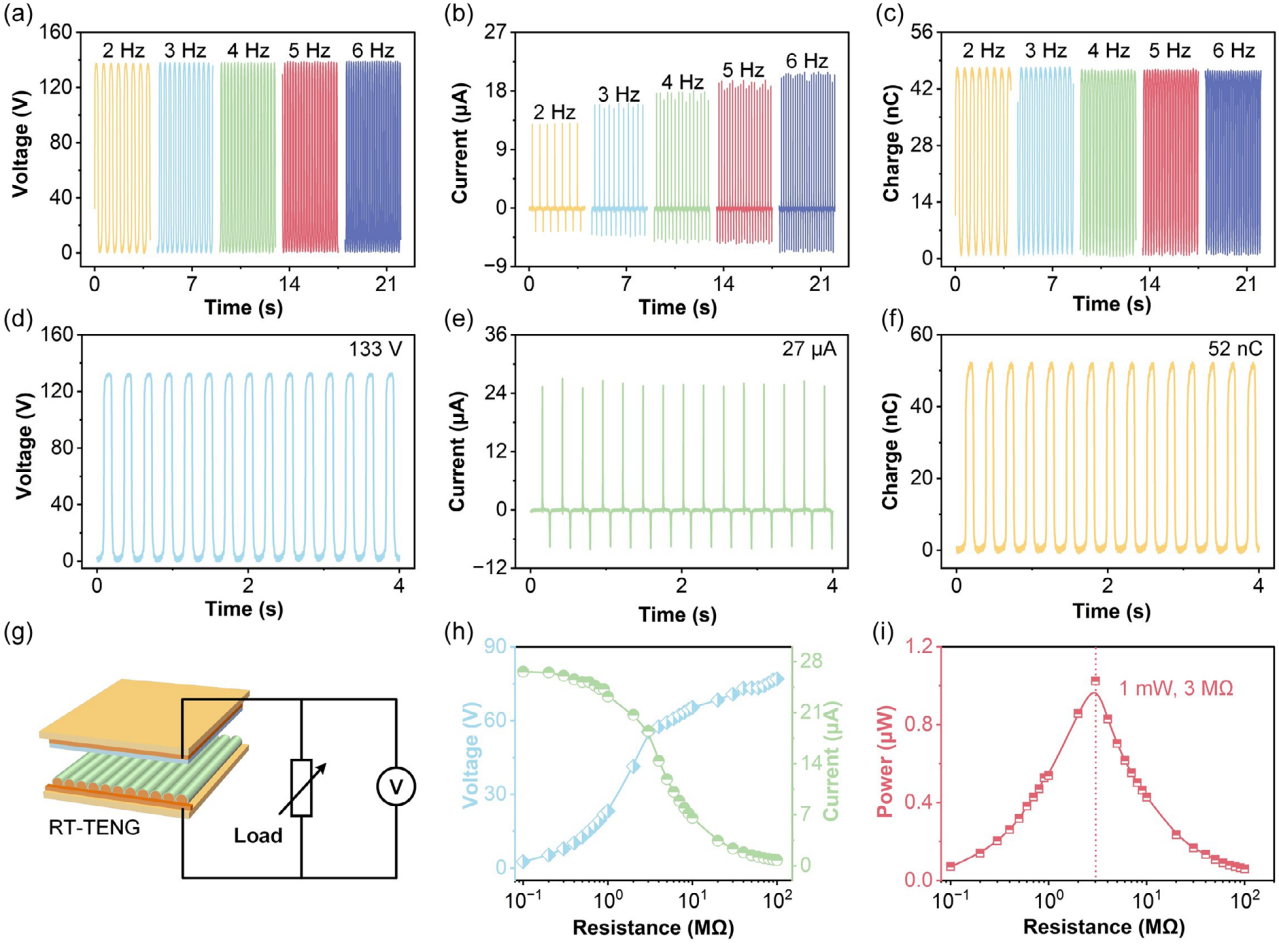
Electrical performance and load‐matching characteristics of the RT‐TENG. (a–c) Output response under different mechanical excitation frequencies. (d–f) Stable electrical signals under continuous periodic operation. (g) Schematic representation of the RT‐TENG connected to an external resistive load for output power evaluation. (h) Dependence of output voltage and current on external load resistance. (i) Output power as a function of load resistance.

Figure 3a shows that the *V*_OC_ remains relatively stable at all frequencies, with slight fluctuations around 136 V. This suggests that the RT‐TENG is robust in terms of voltage generation, unaffected by the frequency changes within this range. In Figure 3b, the *I*_SC_ is shown to increase with frequency, from approximately 12.9 μA at 2 Hz to 20.8 μA at 6 Hz. This increase is attributed to the higher rate of contact‐separation cycles at increased frequencies, which results in a higher current generation rate, demonstrating the frequency‐dependent performance of the RT‐TENG. Figure 3c presents the *Q*_SC_, which remains nearly constant at around 47.1 nC across the frequencies tested. Despite the increase in *I*_SC_, the amount of transferred charge per cycle remains unchanged, indicating that while frequency influences the rate of current generation, it does not affect the total charge transfer per cycle. Figure 3d shows the *V*_OC_ output with a peak value of 133 V, exhibiting well‐defined sinusoidal waveforms, confirming repeatable charge accumulation and release. Figure 3e displays the *I*_SC_ pulses with a maximum amplitude of 27 μA, highlighting the sharp response and low noise, which further illustrates the device's efficiency in capturing and transmitting mechanical energy into electrical signals. Figure 3f shows the *Q*_SC_ reaching up to 52 nC, demonstrating reliable and consistent charge transfer during each cycle, reinforcing the stable triboelectric performance under cyclic loading conditions. Figure 3g shows the circuit configuration for load‐matching measurements, where the RT‐TENG is connected to variable resistive loads, and the voltage across the load is monitored. Figure 3h plots the relationship between external load resistance and the output voltage and current. As the load resistance increases from 0.1 to 100 MΩ, the output voltage increases gradually to approximately 77 V, while the output current decreases from about 26.6 to 0.77  μA. This crossover behavior is indicative of the effect of load impedance on the output signal shape and amplitude, reflecting the trade‐off between voltage and current as the load resistance is adjusted. Finally, Figure 3i presents the calculated output power as a function of load resistance. A peak output power of approximately 1.0 mW is achieved at 3 MΩ, which corresponds to the optimal impedance matching condition for efficient energy transfer. Beyond this optimal load resistance, the output power decreases due to reduced current flow at higher resistance or voltage suppression at lower resistance.

**FIGURE 4:**
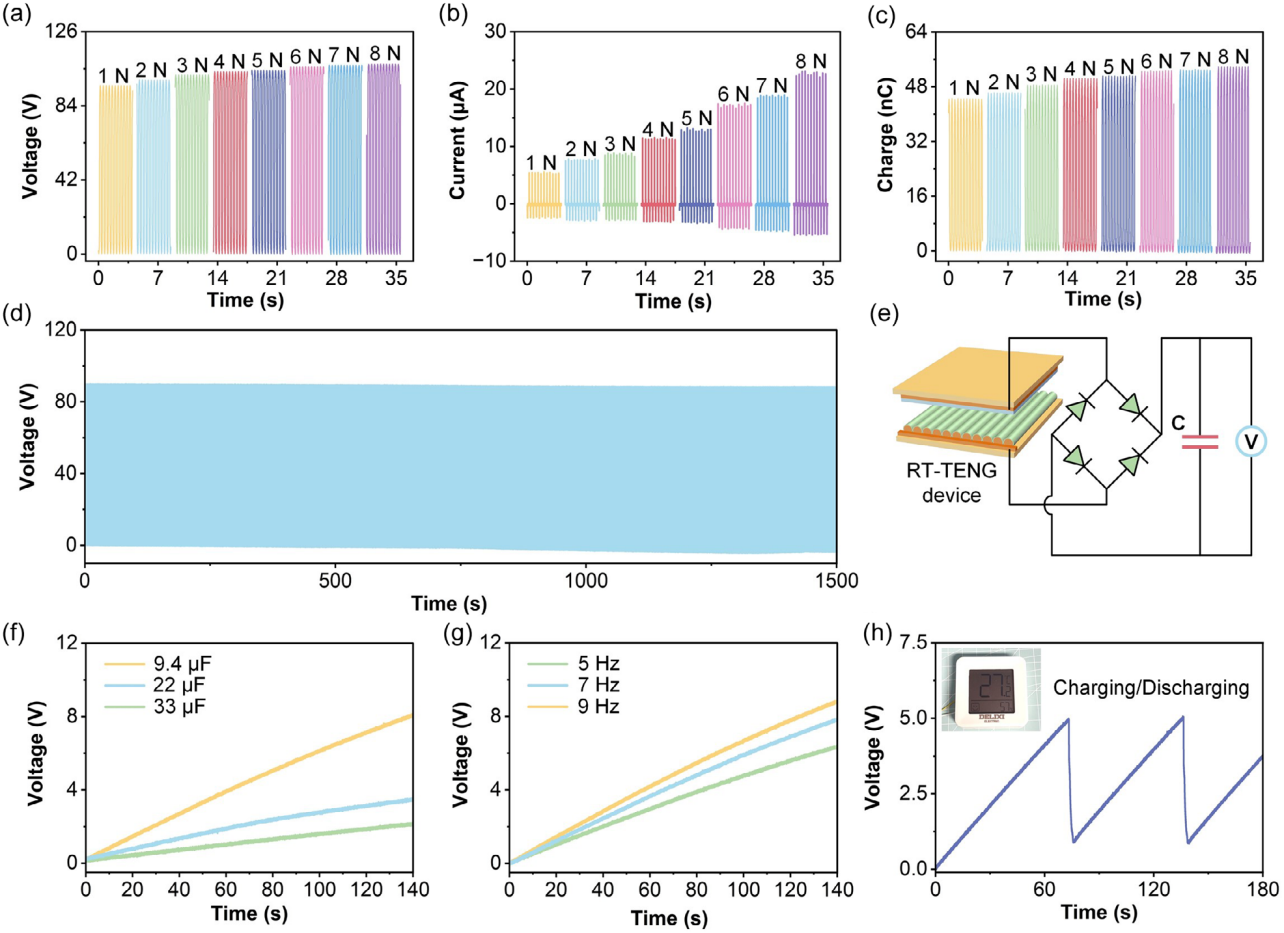
Force dependence, durability, and energy storage performance of the RT‐TENG. (a–c) Electrical output under varying applied forces. (d) Long‐term durability test showing stable V_OC_ during extended cyclic operation. (e) Schematic of the energy storage circuit with a full‐wave rectifier and capacitor. (f) Charging behavior of capacitors with different capacitances under identical excitation. (g) Influence of excitation frequency on capacitor charging rate. (h) Charging and discharging behavior of a capacitor connected to the RT‐TENG; inset shows powering of a commercial electronic device.

Figure 4a–c illustrates the dependence of the electrical output of the RT‐TENG on external applied force. The device was subjected to mechanical stimuli ranging from low to high normal forces. As shown in Figure 4a, *V*_OC_ increases with increasing force, indicating enhanced interfacial contact and more effective triboelectric charge transfer. Figure 4b shows that *I*_SC_ also increases accordingly, reflecting higher instantaneous charge flow rates under larger deformation. In Figure 4c, Qsc exhibits a rising trend, suggesting that greater applied force leads to more significant charge accumulation per cycle due to improved effective contact area and surface deformation. Figure 4d presents the durability and operational stability of the RT‐TENG over extended cycles. The *V*_OC_ waveform remains stable throughout prolonged operation, with negligible signal attenuation, confirming the robustness and mechanical endurance of the device. Figure 4e shows the rectification circuit used for energy storage evaluation, where the RT‐TENG is connected to a full‐wave bridge rectifier and a capacitor. The voltage across the capacitor is monitored to evaluate the energy harvesting capability of the device. Figure 4f shows the charging behavior of capacitors with different capacitances. The voltage accumulation across the capacitor increases gradually over time. Devices with smaller capacitance values exhibit faster voltage rise due to their lower energy storage capacity, while larger capacitors show slower but steady growth, indicating continuous energy delivery from the RT‐TENG. Figure 4g presents the effect of excitation frequency on capacitor charging. Under identical capacitive loads, higher frequencies result in faster charging rates. This is attributed to the increased number of charge generation cycles within a given time, leading to more rapid energy accumulation. Figure 4h demonstrates the charging and discharging behavior of the RT‐TENG when connected to a practical electronic load. The voltage across the storage capacitor exhibits a periodic triangular waveform, indicating repeated energy accumulation and release. The inset shows the successful powering of a commercial digital thermometer, verifying the feasibility of the RT‐TENG for driving low‐power electronic devices.

### 3.4 Wearable Joint‐Motion Monitoring for Taekwondo using the RT‐TENG Device

**FIGURE 5:**
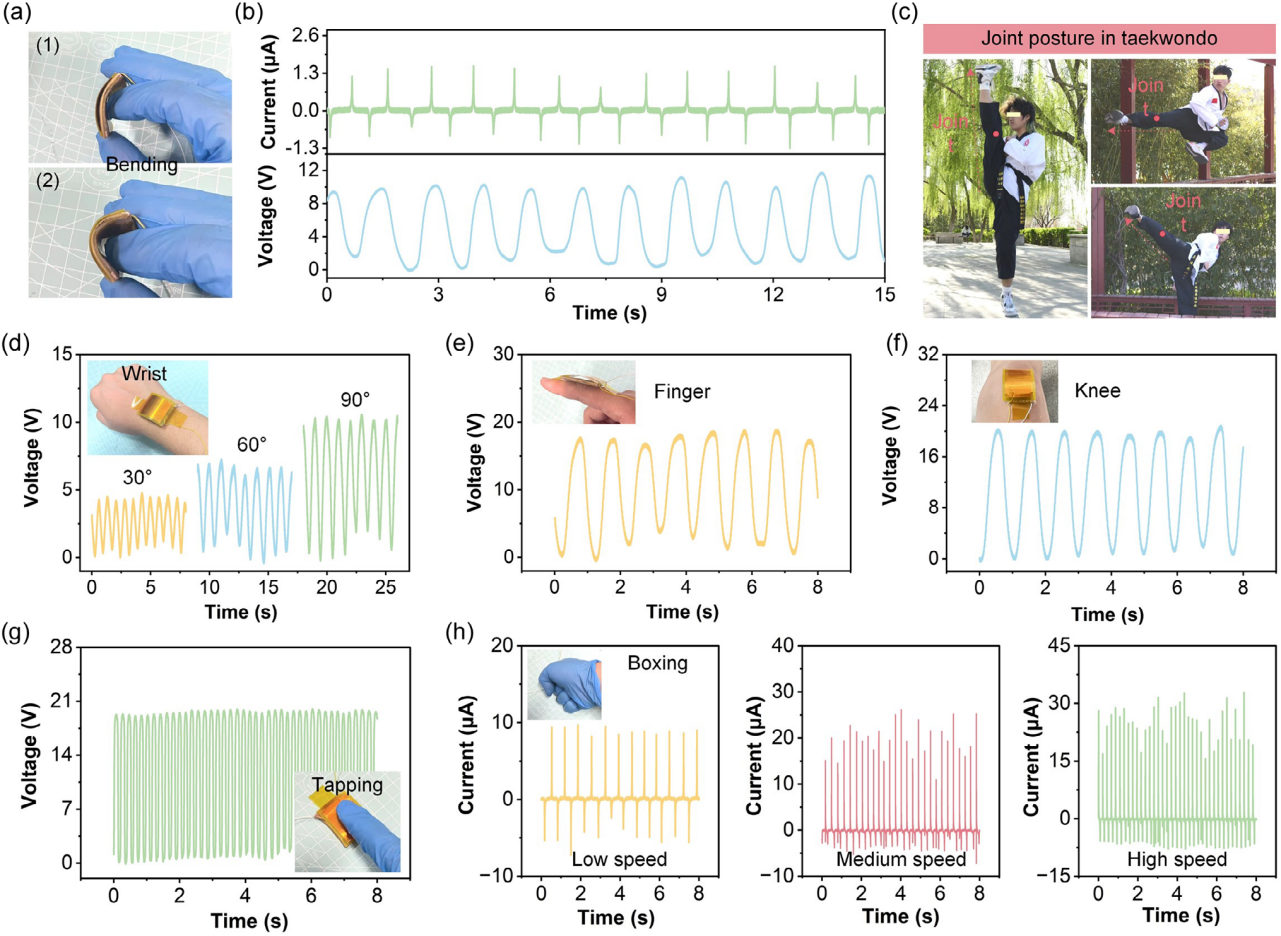
Wearable motion sensing and joint monitoring performance of the RT‐TENG. (a) Photographs of the RT‐TENG under repeated bending. (b) Voltage and current outputs during cyclic bending. (c) Real‐world demonstration of joint posture monitoring during taekwondo movements. (d) Voltage output of the RT‐TENG attached to the wrist under different bending angles (30°, 60°, 90°). (e) Voltage signals generated by finger flexion when the device is mounted on the finger joint. (f) Voltage output corresponding to knee bending. (g) Voltage signals under continuous tapping. (h) Current outputs under boxing‐like impacts at low, medium, and high speeds.

The flexibility and application potential of the RT‐TENG in wearable biomechanical sensing were investigated through bending, tapping, and joint motion monitoring, particularly in the context of taekwondo training and performance assessment. Figure 5a shows the physical flexibility of the RT‐TENG, which can be repeatedly bent without structural damage. Figure 5b presents the voltage and current outputs under cyclic bending conditions. The device generates stable and repeatable electrical signals with well‐defined waveform shapes, indicating its mechanical resilience and sensitivity under low‐strain deformation. To explore its utility in martial arts motion analysis, Figure 5c presents joint monitoring during taekwondo movements, where large joint‐angle variations, particularly in the knee and hip, are critical for technical performance and injury prevention. The RT‐TENG is integrated at joint regions to extract joint‐specific electrical signatures during dynamic postures. Figure 5d demonstrates the voltage response when the device is attached to the wrist and bent to different angles (30°, 60°, and 90°). The signal amplitude increases with greater bending angles, confirming the angular sensitivity of the sensor and its ability to distinguish between fine joint articulation levels. This capability is critical in taekwondo where wrist orientation affects blocking and striking precision. Figure 5e shows the voltage output when the RT‐TENG is mounted on the finger. Periodic finger flexion generates strong and consistent voltage signals, suggesting feasibility for monitoring hand posture, grip dynamics, or finger coordination during forms or weapon handling. Figure 5f illustrates the output signal from the knee during gait‐like flexion. The device records high‐amplitude, periodic voltage peaks corresponding to knee bending cycles, which is essential for assessing lower‐limb kinematics in high‐kick techniques or stance transitions during taekwondo practice. In addition to joint bending, the RT‐TENG also responds to dynamic tapping, as shown in Figure 5g. The voltage signals remain stable and dense under continuous tapping, indicating rapid mechanical response and potential for impact detection, such as foot strikes or punch landing assessment. Figure 5h further evaluates current output under boxing‐like impacts at different speeds. At low speed, the current peaks are well‐separated and consistent, while medium and high‐speed strikes result in higher frequency and amplitude signals with less spacing between events. This demonstrates the RT‐TENG's capability to resolve variations in movement speed and impact intensity. These results validate the RT‐TENG as a multifunctional wearable sensor platform for detecting diverse joint motions and dynamic mechanical inputs. Its ability to track wrist, finger, and knee movement with high fidelity supports its integration into taekwondo performance monitoring systems. By capturing continuous biomechanical data from joint activity, the RT‐TENG can assist athletes and coaches in quantitatively evaluating form accuracy, motion range, and strike quality. Moreover, the sensor's lightweight structure, flexibility, and self‐powered operation make it suitable for long‐term use in wearable training gear without the need for external power sources. This paves the way for real‐time, on‐body biomechanical monitoring in martial arts and other high‐intensity sports.

## 4 Conclusions

In summary, we designed and demonstrated a RT‐TENG based on a tube architecture, aimed at harvesting biomechanical energy and enabling motion sensing in intelligent sports applications. Distinct from conventional multilayered structures, the integrated design combines the triboelectric layer, electrode, and support framework into a single tubular unit, streamlining the fabrication process and reducing manufacturing costs without compromising output stability. Using FEP and as the triboelectric materials, the device exhibits strong adaptability to environmental conditions, with enhanced output under elevated temperatures and signal attenuation under high humidity. Performance evaluation shows a peak *V*_OC_ of 133 V, *I*_SC_ of 27 μA, and *Q*_SC_ of 52 nC, alongside a maximum output power of approximately 1.0 mW at 3 MΩ load resistance. The RT‐TENG also exhibits robust mechanical durability and reliable energy storage capability and is capable of driving a digital thermo‐hygrometer, verifying its practicality in self‐powered electronics. Furthermore, its sensitivity to joint movement and impact intensity during taekwondo demonstrates its potential as a flexible, wearable platform for high‐fidelity biomechanical monitoring in dynamic sports environments.

## Author Contributions

**Chengquan Piao**: conceptualization (lead), data curation (lead), formal analysis (lead), funding acquisition (lead), investigation (lead), methodology (lead), project administration (lead), resources (lead), software (lead), supervision (lead), validation (lead), visualization (lead), writing – original draft (lead), writing – review and editing (lead). **Jianzhong Wan**: conceptualization (equal), data curation (equal), formal analysis (equal), funding acquisition (equal), investigation (equal), methodology (equal), project administration (equal), resources (equal), software (equal), supervision (equal), validation (equal), visualization (equal), writing – original draft (equal), writing – review and editing (equal). **Qichen Mu**: conceptualization (equal), data curation (equal), formal analysis (equal), funding acquisition (equal), investigation (equal), methodology (equal), project administration (equal), resources (equal), software (equal), supervision (equal), validation (equal), visualization (equal), writing – original draft (equal), writing – review and editing (equal). **Peiying Jia**: conceptualization (supporting), data curation (supporting), formal analysis (supporting), funding acquisition (supporting), investigation (supporting), methodology (supporting), project administration (supporting), resources (supporting), software (supporting), supervision (supporting), validation (supporting), visualization (supporting), writing – original draft (supporting), writing – review and editing (supporting).

## Conflicts of Interest

The authors declare no conflicts of interest.

## Data Availability

Data available on request from the authors.
